# Fluctuating salience in those living with genetic risk of motor neuron disease: A qualitative interview study

**DOI:** 10.1111/hex.14024

**Published:** 2024-03-25

**Authors:** Jade Howard, Fadhila Mazanderani, Karen Forrest Keenan, Martin R. Turner, Louise Locock

**Affiliations:** ^1^ Division of Neuroscience, Sheffield Institute for Translational Neuroscience University of Sheffield Sheffield UK; ^2^ School of Social and Political Science, Science, Technology and Innovation Studies University of Edinburgh Edinburgh UK; ^3^ Epidemiology Group University of Aberdeen Aberdeen UK; ^4^ Nuffield Department of Clinical Neurosciences University of Oxford Oxford UK; ^5^ Health Services Research Unit University of Aberdeen Aberdeen UK

**Keywords:** amyotrophic lateral sclerosis, genetic risk, inherited, interviews, motor neuron disease, qualitative

## Abstract

**Background:**

Motor neuron disease (MND) (also known as amyotrophic lateral sclerosis) is a life‐limiting neurodegenerative condition. In up to 20% of people with MND, a pathogenic variant associated with autosomal dominant inheritance can be identified. Children of people carrying a pathogenic variant have a 50% chance of inheriting this and a higher, although harder to predict, chance of developing the disease compared to the general adult population. This paper explores the experience of living with the genetic risk of MND.

**Methods:**

We undertook a UK‐based interview study with 35 individuals, including: 7 people living with genetically‐mediated forms of MND; 24 asymptomatic relatives, the majority of whom had an increased risk of developing the disease; and 4 unrelated partners.

**Results:**

We explore how individuals make sense of genetic risk, unpacking the interplay between genetic knowledge, personal perception, experiences of the disease in the family, age and life stage and the implications that living with risk has for different aspects of their lives. We balance an emphasis on the emotional and psychological impact described by participants, with a recognition that the salience of risk fluctuates over time. Furthermore, we highlight the diverse strategies and approaches people employ to live well in the face of uncertainty and the complex ways they engage with the possibility of developing symptoms in the future. Finally, we outline the need for open‐ended, tailored support and information provision.

**Conclusions:**

Drawing on wider literature on genetic risk, we foreground how knowledge of MND risk can disrupt individuals' taken‐for‐granted assumptions on life and perceptions of the future, but also its contextuality, whereby its relevance becomes more prominent at critical junctures. This research has been used in the development of a public‐facing resource on the healthtalk.org website.

**Patient or Public Contribution:**

People with experience of living with genetic risk were involved throughout the design and conduct of the study and advised on aspects including the topic guide, sampling and recruitment and the developing analysis. Two patient and public involvement contributors joined a formal advisory panel.

## 1 INTRODUCTION

Advances in genetic knowledge and technologies have shifted understandings on the causes of many conditions. This is the case in motor neuron disease (MND), also known as amyotrophic lateral sclerosis. This neurodegenerative disease leads to progressive weakness of muscles and can affect mobility, speech and breathing, as well as wider brain involvement impacting cognition and behaviour. The typical survival from symptom onset is 2–5 years.^1^ It is predicted that increasing numbers of people with MND will access genetic testing, a consequence of widening eligibility criteria and genetically targeted clinical trials (with one genetically targeted therapy suggesting clinical benefit).^2^, ^3^, ^4^ This means more family members will become aware of their increased risk of developing the condition.

The genetics of MND are complex,^5^ but it is suggested that in up to 20% of people with MND, a pathogenic variant can be identified.^6^ Since the identification of *SOD1* over two decades ago, pathogenic variants in more than 25 genes linked to inherited forms of MND (iMND) have been identified, with some (including a hexanucleotide repeat expansion in *C9orf72*) linked to frontotemporal dementia (FTD), which has clinical and pathological overlap with MND. There are currently around one‐third of individuals with an autosomal dominant family history of MND for whom no genetic variant can be identified.^7^

Most inherited forms of MND are associated with autosomal dominant inheritance, meaning children of people carrying a pathogenic variant have a 50% chance of inheriting this, which can be confirmed through predictive genetic testing (also known as presymptomatic testing). However, penetrance (the chance an individual carrying a pathogenic variant will go on to develop symptoms) is variable across different gene variants and families. Therefore, it is not possible to predict penetrance for an asymptomatic individual, what age symptoms will first show themselves, or how they will develop.^5^, ^8^ Reproductive options, including preimplantation genetic testing, are potentially available for those wishing to reduce the risk of iMND in future generations.^9^, ^10^

The experiences of individuals with an increased genetic risk of MND have received little research attention and none in the United Kingdom. Exceptions include several US‐based studies. Fanos et al.^11^ found around half of untested participants reported anxiety about living with risk, which manifested in risk‐taking behaviour, intrusive thoughts and trouble sleeping. Psychological impacts included guilt around siblings being affected or anticipatory guilt of burdening children. At the same time, people tried to take positives from their situation, finding motivations to live life to the fullest. Positive changes were also reported in a study on individuals from families affected by *SOD1*‐linked MND, with a variety of genetic testing decisions and outcomes.^12^ Here, people reported a motivation to take care of their health, clearer priorities and perspectives, and changed life decisions. Nonetheless, some suggested that concerns over developing the disease escalated over time, including concerns around the end of life.^12^ Hartzfeld et al.^13^ similarly highlight the balance of positive and challenging aspects of living with risk, as individuals reported personal and family strains as a result of their experiences, alongside stronger relationships, altered priorities and a renewed appreciation for life. Most recently, Dratch et al.^14^ noted identity implications in individuals who tested positive for gene variants linked to iMND/FTD. Experiences were characterised by uncertainty, dread and anticipatory grief over future losses, which could impact people's lives and self‐perceptions. People accepted, rejected and integrated knowledge of their increased risk into their identities in many ways, with participants describing this knowledge as positive and enabling, unique or isolating.

While studies have explored experiences of genetic risk in other conditions, it is worth noting the characteristics of MND that underscore the need for research amongst this population. Unlike certain hereditary cancers where there may be options to screen, treat or prevent disease, MND treatment options are limited.^11^ However, clinical trials are ongoing and there is now a gene therapy, tofersen, licensed in the United States to treat patients with certain *SOD1* gene variants, with a presymptomatic trial underway.^15^ While Huntington's disease (HD) is another incurable, progressive, neurodegenerative disorder, iMND is distinguished from this latter condition by an incomplete understanding of its genetic architecture.^7^, ^16^ Further, the variable penetrance of MND‐linked genes may impact the perceived utility of genetic testing for at‐risk individuals.^13^ Indeed, Crook et al.^16^ suggest families affected by iMND face more uncertainty and complexity than in other neurodegenerative conditions. Given how meanings around genetic risk are shaped by the particular characteristics of the disease,^17^ this study responds to a need for research amongst this population.^8^, ^12^, ^13^, ^18^

Funding was secured to use interviews carried out for this study to develop a new resource on the healthtalk.org website. This is a web‐based resource that provides a source of information and support on iMND, based on lived experience and illustrated with video, audio and written clips from interviews. Published in June 2022, it can be found at: https://healthtalk.org/introduction/inherited-motor-neurone-disease-mnd/.

This study aimed to explore the following questions: how do people make sense of being at risk of MND? How do they experience living with risk over time? And what impact does this knowledge have across aspects of their lives and decisions?

## 2 MATERIALS AND METHODS

This paper is based on a UK‐based interview study on family experiences of iMND. Ethics approval was granted by the Berkshire Ethics Committee (REC Ref 12/SC/0495). Of the 35 participants, the majority (*n* = 24) were asymptomatic family members of people with suspected or confirmed iMND. Of these, six had tested positive for an MND‐linked gene variant, three had tested negative and one additional person had been determined to be negative following her parent's negative result. The remaining 14 did not know their genetic status. In some families, a genetic cause of the disease had not been identified, but they understood it to be hereditary based on family history. The other participants were people with MND who identified as having an inherited form (*n* = 7) and partners of people living with MND/genetic risk (*n* = 4). Participants were aged from 24 to 69, with 22 female and 13 male. A higher proportion of female participants is a pattern reflected in other qualitative studies.^19^ One individual identified as mixed ethnicity and the remainder as White/White British. Seeking additional participants from ethnic minority backgrounds through targeted recruitment was unsuccessful, perhaps in part due to the rarity of the condition.

Recruitment was carried out through multiple avenues (Table 1). Snowball sampling enabled the recruitment of individuals who did not typically engage with research or the MND charities. A maximum variation sampling approach^20^ was used with the aim of including people with diverse genetic testing decisions and outcomes, and at different life stages (see Tables 2, 3, 4 for participant details).

**Table 1:** Interview participant recruitment details.

Recruitment avenue	Study details shared through:	Participants recruited
Families for the Treatment of Hereditary MND initiative	Presentation at meeting day; Facebook support group	7
MND Association	Website; blog post; newsletters (including research mailing list); Twitter	15
MND Scotland	Website; support groups	2
Oxford MND centre	Distribution of information packs to interested individuals attending the clinic who met criteria for participation	1
The Euan MacDonald centre (Edinburgh‐based research centre)	Website; blog post; social media	4
Other virtual peer support groups	Facebook groups	0 (all participants who got in touch were based outside the United Kingdom)
Snowball sampling	Details shared through existing participants	6

Abbreviation: MND, motor neuron disease.

**Table 2:** Participant characteristics: People with an increased risk of developing MND.

ID.	Pseudonym	Sex	Age	Gene variant in the family	Predictive genetic testing status	Children
1	Jen	F	50–59	*C9orf72*	Not tested	Yes
3	Fiona	F	40–49	*SOD1*	Not tested	No children
5	Jackie	F	40–49	Unknown	Not tested	Yes
6	Greg	M	40–49	Known but cannot remember	Not tested	Yes
7	Maria	F	50–59	*C9orf72*	Tested—positive	No children
8	Elaine	F	50–59	Unknown	Not tested	Yes
9	Ricky	M	40–49	*C9orf72*	Tested—positive	Yes
10	Thomas	M	40–49	*C9orf72*	Not tested	Yes
15	Aaron	M	20–29	*C9orf72*	Tested–positive	No children
17	Marion	F	60–69	*C9orf72*	Tested–negative	No children
19	Beverley	F	50–59	*C9orf72*	Tested—positive	Yes
20	Siobhan	F	30–39	SOD1	Not tested	Yes
21	Jasmine	F	20–29	*C9orf72*	Not tested	No children
22	Oscar	M	20–29	*C9orf72*	Not tested—undergoing genetic counselling	No children
23	Dean	M	20–29	Unknown	Not tested	No children
24	Gordon	M	40–49	Unknown	Not tested	Yes
26	Susan	F	50–59	Unknown	Not tested	Yes
29	Alex	Withheld	Withheld	*C9orf72*	Not tested—undergoing genetic counselling	Withheld
31	Stacey	F	30–39	*C9orf72*	Tested—negative	No children
32	Anna	F	30–39	*C9orf72*	Tested—positive	Yes
33	Rachael	F	30–39	*C9orf72*	Not tested—confirmed negative through parent's genetic test	No children
34	Claire	F	30–39	*C9orf72*	Not tested	Yes
35	Steph	F	30–39	*C9orf72*	Tested—positive	Yes
36	Sophie	F	20–29	*C9orf72*	Tested—negative	No children

Abbreviations: F, female; M, male; MND, motor neuron disease.

**Table 3:** Participant characteristics: People with MND.

ID.	Name	Sex	Age	Gene variant in the family	Genetic testing status	Children
2	Oliver	M	60–69	*C9orf72*	Tested	No children
12	Ian	M	60–69	Inconclusive	Tested	Yes
13	Debbie	F	50–59	*C9orf72*	Tested	Yes
16	Anya	F	60–69	*SOD1*	Tested (gene variant already identified in family member)	Yes
18	Eric	M	60–69	*C9orf72*	Tested	Yes
25	Caroline	F	60–69	No gene variant identified	Tested	Yes
30	Mark	M	60–69	No gene variant identified	Tested	Yes

Abbreviations: F, female; M, male; MND, motor neuron disease.

**Table 4:** Participant characteristics: Partners.

ID.	Name	Sex	Age	Gene variant in partner's family	Children	Other details
4	Mairi	F	60–69	Did not disclose	Yes	Partner of pwMND
11	Julie	F	40–49	*C9orf72*	Yes	Partner of person at risk
14	Arthur	M	60–69	*C9orf72*	Yes	Partner of pwMND
27	Diane	F	60–69	Inconclusive	Yes	Partner of pwMND

Abbreviations: F, female; M, male; pwMND, people with motor neuron disease.

Potential participants who expressed interest in the study were contacted using their preferred method (phone, email, etc.) to discuss the study and given the chance to ask questions. Consent was taken before each interview. Interviews followed an intensive interviewing approach,^21^ with a topic guide used flexibly to guide discussion, based on literature reviews and a study of posts related to iMND from the MND Association forum.^22^ The topic guide consisted of open‐ended questions, covering diverse aspects anticipated to be relevant to living with iMND, including the discovery of iMND in the family; knowledge, information and support; genetic testing; reproductive choices and family communication. The topic guide was adapted depending on participant characteristics and evolved over the interviews.^21^

The majority of the 11 face‐to‐face interviews were carried out in people's homes or another place of their choosing; the remaining 24 were conducted remotely due to the Covid‐19 pandemic. Interviews lasted an average of just under 2 h. They were audio recorded and transcribed verbatim by a professional transcriber. Where permission was obtained, video recording was carried out for the purpose of developing the aforementioned resource on healthtalk.org. Interviews were deemed to hold sufficient ‘information power’^23^ to stop recruitment at 35 participants, on the basis that the research ‘offers new insights that contribute substantially to or challenge current understandings’.^23^ ^,p.1759^

Transcripts were checked, anonymised and returned to participants who could make changes or omissions. Approved transcripts were coded by J. H. following a constructivist grounded theory‐informed approach^21^ and facilitated by Nvivo. Initial inductive coding was followed by focused coding where pertinent codes were interrogated and tested until it was deemed the developing framework best encapsulated the data. All themes were unpacked and analysed through the OSOP (‘One Sheet of Paper’) method where coding reports were visually mapped and the relationship between themes and subthemes developed.^24^ A constant comparison approach was used and memos were recorded throughout. ‘Experiences of living with knowledge of genetic risk’ was one of several themes generated, with this paper presenting four subthemes of this broader theme: making sense of being at risk; the emotional and psychological impact of risk; the fluctuating salience of risk and living well while preparing for the future.

People with experience of genetic risk (patient and public involvement [PPI] contributors) were invited to express interest in advising the study at a meeting day for families affected by MND. PPI contributors were involved throughout the design and conduct of the study, giving feedback on aspects including the topic guide, sampling and recruitment and the developing analysis. Two PPI contributors joined a more formal advisory panel that provided advice and oversight over the project.

Pseudonyms have been used throughout for anonymity. Participant quotations are accompanied by details to indicate age and participant group. Relatives are grouped by predictive testing status (‘tested positive’, ‘tested negative’ and ‘not tested’). Quotations have been lightly edited for readability.

## 3 RESULTS

### 3.1 ‘Doing the maths’: Making sense of being at risk

Making sense of genetic risk was an ongoing process. Most participants understood the chance of inheriting MND‐linked gene variants as ‘50:50’, reflected in metaphors such as ‘the toss of a coin’ (quotation 1, Table 5). Quantifying risk was complicated where the visible pattern of the disease in the family did not appear to represent the principles of autosomal dominant inheritance. One participant calculated how many people in each generation of her family had been affected, using this genealogical research to make sense of her own risk (quotation 2, Table 5).

**Table 5:** Illustrative quotations for theme ‘“Doing the maths”: making sense of being at risk’.

Quote no.	Illustrative quotation
1	It's 50:50, you can't feel it in yourself, it's not like going for a test for cancer, if you've got a lump … I didn't even let myself think that way because it's illogical, you can't predict. (Maria, 50–59; tested positive)
2	It doesn't really seem to make much sense … my great grandpa was one of 12 so he had a one in 12 chance, and then my grandfather's one in six, so basically they had one in three because two of them got it. My dad's one in four, so I really don't know to be honest with you and I don't actually want to know. (Siobhan, 30–39; not tested)
3	I've always had … a feeling deep inside, that I probably will get it, only because I was really close to him [father] and I just feel that we were somehow alike, more alike than what my sister was to him. And then my grandma … I thought then it might be passed down because I had some gene because of her eyes. Just silly stuff like that. (Jackie, 40–49; not tested)
4	My daughter … she's like a carbon copy of me … I thought, ‘Oh my god, she's going to have this gene,’ but I have to remind myself that it is 50:50. (Steph, 30–39; tested positive)

Knowing the statistical chance of inheritance did not mean everyone experienced risk in these terms. Participants sometimes described having a ‘feeling’, ‘sense’ or ‘hunch’ about whether they or other relatives would be affected. Likeness to affected relatives (physical and personality characteristics), as well as patterns of the disease in terms of sex and birth order fed into sense‐making. Some saw themselves as like both parents, upholding a 50:50 risk. Seeing herself as ‘more like my dad’, Sophie (20–29; tested negative), who lost her mum to MND and FTD, perceived a lessened risk before having genetic testing. Jasmine's (20–29; not tested) assessment that the family had had enough ‘bad luck’ underlay her hope that she and her mum might ‘be spared’. A more common view, however, was that of having a heightened risk (quotation 3, Table 5), which some suggested was a form of coping.

Personal risk was made sense of in relation to perceptions about siblings' risk. One individual recalled asking the consultant whether the babies her mother had miscarried could have been affected, meaning she might have had a ‘lucky escape’, evoking a sense of risk being spread between siblings rather than the coin being tossed anew each time. Marion (60–69; tested negative) could not imagine testing negative, as her siblings who had been tested before her all carried the *C9orf72* gene variant: ‘It just seems too many … This 50:50 isn't 50:50 at the moment, it's 100%’. Her daughter Rachael described perceiving an ‘extra strong gene’, while acknowledging there was ‘no scientific reasoning for this’. Indeed, participants sometimes acknowledged their feelings and hunches were not necessarily ‘scientific’, with some people reminding themselves that the possibility of inheritance was 50:50, particularly in reference to their own children (quotation 4, Table 5).

People were often aware that not everyone who carries a gene variant will develop symptoms, a source of hope across interviews. Interviewees gave various statistics about the penetrance of MND‐linked gene variants, from ‘1/3’, ‘50:50’ to ‘100%’, and some understood risk to increase with age. Anna (30–39; tested positive) vividly recalled her neurologist telling her it was ‘more likely than not’ she would develop symptoms after her positive predictive test, a sentence which ‘has stayed etched in my brain’. Some had been informed by healthcare professionals that penetrance could not be accurately estimated. Participants sometimes expressed hopes of avoiding ‘turning on’ or ‘triggering’ the gene, though there was an awareness that factors contributing to disease onset are not well understood.

People often imagined they would develop symptoms around the age relatives had been affected and talked about ‘doing the maths’ to calculate how long they might have before this point. Alex (age withheld; not tested) based calculations on the family history and knowledge of the average age of onset: ‘I'm now sort of planning towards the median age for onset, just in case … leave my partner in good shape’.

### 3.2 ‘A sword hanging over your head’: The emotional and psychological impact of risk

Living with risk affected each participant differently. A few individuals described feeling able to keep worries in the background; MND risk did not exert a significant influence in their everyday lives. Dean (20–29; not tested) described: ‘I don't sit and worry about what could happen, it's kind of like if anything happens, I'll deal with it…’

However, for the most part, participants described knowledge of genetic risk as having some level of emotional or psychological impact, affecting their sense of self and perceptions of the future. Participants mentioned periods of feeling hopeless, afraid, anxious or depressed, experiencing panic or having trouble sleeping (quotation 1, Table 6). Some people described feelings of futility and questioning the point of going on (quotation 2, Table 6).

**Table 6:** Illustrative quotations for the theme ‘“A sword hanging over your head”: the emotional and psychological impact of risk’.

Quote no.	Illustrative quotation
1	There's the sense of doom, hopelessness, anxiety, I think it's overwhelming, you know? It's a very existential situation. Everyone has to accept that they're going to die, but with this disease, there's a certain route it takes. (Oscar, 20–29; not tested)
2	I did particularly a few years ago, let's say I would have had a lot of thoughts about things being quite futile … there is a bit of that in the back of your mind … I have struggled with suicidal thoughts and that kind of thing. (Gordon, 40–49; not tested)
3	This test has changed my thought processes about certain things and it made me a little bit more cautious. But at the same time we've still done some really bonkers things because we've only got one life. It's made me value life better. (Beverly, 50–59; tested positive)
4	It gave me a loss of what might be, it gave me a loss of maybe being a grandmother perhaps, it gave me a loss of growing old and grumpy with my husband perhaps, it gave me a loss of some of my own sense of self. (Beverly, 50–59; tested positive)
5	How do you tell somebody that they can't have a child naturally with you … when there's that much choice out there for other people that don't have these problems, I think you feel very vulnerable that people will walk away from you. (Aaron, 20–29; tested positive)
6	Of greater concern to me is the risk I've passed it to my children, and if they have children, my grandchildren. If there's a worry for me, it's that. (Greg, 40–49; not tested)
7	The other flip of the coin is that you could never get any of it, so if you're worrying about something that you're not going to get like it's just ridiculous, it just feels horrible. (Aaron, 20–29; tested positive)

Claire (30–39; not tested) recalled being more ‘reckless’ after discovering a genetic cause of her mother's condition, drinking more regularly and being less thoughtful about spending money. This only lasted a few weeks until she thought to herself ‘Don't be stupid, sort your life out’. Nonetheless, the grief of her mother's illness and becoming aware of her risk affected her confidence and she described feeling like ‘a lesser person than myself’ at times. Receiving a positive predictive test result could also have a distinct impact. Aaron still had a desire to live and achieve his goals, but was more willing to take risks (such as speeding) and no longer valued his life as he did before he knew he carried the *C9orf72* gene variant. An alternative experience, however, was valuing life more and being more cautious (quotation 3, Table 6).

For participants, living with the risk of MND meant grappling with the possibility that they might not have future experiences they had imagined or be around to fulfil family roles (as a parent and grandparent). Although these worries were expressed by those who did not know their genetic status, they could be particularly prominent following a positive genetic test result. Beverley articulated ‘a loss of what might be’ after finding out she carried the *C9orf72* gene variant (quotation 4, Table 6).

For younger people, the risk of iMND could feel like a barrier to experiencing life stages or milestones, including meeting partners, getting married and having children. Aaron worried that meeting someone new would be difficult if his current relationship were to end given his desire to prevent future children from inheriting the *C9orf72* gene variant (quotation 5, Table 6). People also expressed fears over living and dying with the disease, often based on memories of affected relatives. The possibility that other members of the family could have inherited the MND‐linked gene variant was a source of anxiety, distress, and guilt for participants, particularly parents (quotation 6, Table 6).

Uncertainty was a defining aspect of living with risk, both for those with and without knowledge of their personal genetic status. Elaine (50–59; not tested) did not know the genetic variant responsible for the disease in her family. For her, ‘It's really a wait and see, which can be a bit like a sword hanging over your head’. However, worrying about something that may never happen was also a difficult prospect (quotation 7, Table 6). At the same time, the possibility they might not develop symptoms, or if they did it could be later in life, or when effective treatments had been developed, underscored the sense of hope expressed in interviews. Ricky (40–49; positive result) articulated, ‘You have to be positive in life … and not dwell on something that may or may not happen’.

### 3.3 ‘It comes and goes in waves’: The fluctuating salience of risk

The impact of iMND was described as something that is ‘always in the back of your mind’ or ‘lurking in the background’. However, its prominence ebbed and flowed over time, as articulated by Julie (40–49; partner): ‘It comes and goes in waves … it's always on our minds, but it's often hidden away and then it occasionally rears its ugly head’.

People described diverse ‘trigger points’ where worries came to the forefront (quotations 1 and 2, Table 7). People were reminded of their risk when they experienced unusual sensations, particularly those they recognised from relatives. Participants described ‘symptom searching’ and hypervigilance over changes that could indicate the start of the disease; MND became ‘the go‐to place’ when noticing something ‘not quite right’. Some described going into ‘full scale panic’ or ‘meltdown mode’ when this happened (quotation 3, Table 7). Others came to recognise alternative causes for the symptoms and calmed themselves. Being assessed by healthcare professionals (such as physiotherapists or neurologists) helped ease people's worries, though it could be difficult when individuals felt that their legitimate fears were dismissed or not understood, both by those around them and professionals (quotation 4, Table 7). People also reported watching family members for symptoms, including partners, children and grandchildren (quotation 5, Table 7).

**Table 7:** Illustrative quotations for theme ‘“It comes and goes in waves”: the fluctuating salience of risk’.

Quote no.	Illustrative quotation
1	It sort of sits there, ‘this could affect you any day’. Some days are better than others, and I manage it very well, I think for me … my older cousin died last year, and that again set me into a swirl of worry. Going to [research study] … if I fall over something I'm immediately into full scale panic, I think I always will be… It isn't like a tombstone hanging over me, it's like a rollercoaster of ‘it's not really on my mind—it's on my mind—it's not really on my mind’. (Maria, 50–59; tested positive)
2	Whenever there's a high‐profile diagnosis… Mum's birthday, Mother's Day's coming up, anniversary of my mum dying … it's back at the forefront of my mind … On the train to work thinking have I got MND, or not? Push it back into that space that says, ‘No, don't let this rule your life. It is what it is, let's stay positive’. (Thomas, 40–49; not tested)
3	I rationally know in my head that this is not going to hit me until I'm in my 60s and I pray and hope that by then there will be a pill I can take … But it doesn't stop me, every time I trip over and every time I drop something [tearful] feeling like, ‘Am I an anomaly and are these symptoms starting?’ … those fleeting moments are rather debilitating because they do make you just stop and go, ‘Oh, fuck’. (Anna, 30–39; tested positive)
4	I went to the GP last year and I said, ‘I've got fasciculations in my hand,’ and she looked at it and she said, ‘No, there's nothing to worry about,’ but for me, there is something to worry about. And I don't think people understand that overwhelming fear. (Elaine, 50–59; not tested)
5	Every single day I think about it and wonder if my children, if they walk in the door, I'm looking to see if they're limping. (Mairi, 60–69; partner)
6	It became real. Up until that point it was just phone calls and meetings and people saying they were going to take my blood but it was not happening, and then all of a sudden … ‘Oh my god, they're being tested as we speak’ … that was when it hit me, ‘Oh my god …. am I literally going to get ten years with my kids?’ (Stacey, 30–39; tested negative)
7	It's a fly in your glass of wine. It's just a little tiny annoyance. I know it's there, I can fish it out and put it out of the glass if I want to, but it will keep coming back because it's not going away. But there's more wine than there is fly, so it's okay. (Steph, 30–39; tested positive).
8	I feel quite at peace with the thought of it all … but then on the flip side I resent having to go through IVF, 100% … that's frustrating and disappointing but it is what it is. (Anna, 30–39; tested positive)
9	Me and my husband basically put everything on hold, we stopped looking at houses, we stopped talking about children …. when someone tells you there's a very high chance at this moment in time that your life isn't going to go the way you want, you've kind of got to take a step back. (Stacey, 30–39; tested negative)
10	For the last 40 years I've lived with the possibility of developing it at some point. And because both my mum and her mother died at the same age, the years from 50 onwards, I was living in dread of symptoms and every twitch … over the years, it's become much more of an influence on my mental health. (Elaine, 50–59; not tested)
11	I'm [past age where relatives were affected] now so maybe I haven't got it [laughs]. There's no sense in that at all because I could just as easily develop symptoms tomorrow, but I suppose you can only be frightened of something for so long. And then you just have to move on. (Marion, 60–69; tested negative)
12	You think about my mum's situation and then you go into how this might impact yourself, and I do try and push it to the back because obviously things are going on with my mum, but …. I do probably think about it at the moment every time I speak to mum and dad, which is every day. (Claire, 30–39; not tested)

Abbreviations: GP, general practitioner; IVF, in vitro fertilization.

Predictive testing decision‐making and going through the genetic counselling and testing process also pushed thoughts of the disease to the forefront. Sophie (20–29; tested negative) described, ‘I can't believe how much I thought about it … once I knew I could get the testing done, it was like a ticking time bomb, “When's my appointment?”’ Waiting for results was often a period of heightened anxiety, anticipation, and hope. For some, the implications of what it could mean for their future hit home (quotation 6, Table 7). Receiving results, whatever the outcome, was accompanied by a range of emotions. The time after receiving a positive result could be particularly difficult. Maria (50–59; tested positive) felt ‘at sea’ and struggled to find support. However, she did not think this additional knowledge had changed her level of worry overall. Longer term, participants generally felt they could process the result and get on with their lives, although with various ongoing impacts (quotation 7, Table 7). People who had tested negative pointed out that MND would continue to be a part of their lives through loss or risk to other relatives.

For some participants, risk became prominent around reproduction. After finding out she carried the *C9orf72* gene variant, Anna and her partner decided to try pre‐implantation genetic testing using IVF to expand their family. It was the reproductive implications that she foregrounded when describing the impact of her risk (quotation 8, Table 7). Certain younger participants had also considered MND risk in this context. Some indicated that until they knew their genetic status, risk was at the front of their minds and presented a barrier to moving forwards with their plans and goals (quotation 9, Table 7). Others recalled having considered MND risk at the time of making reproductive choices, with various outcomes (with participants proceeding to have children or deciding not to expand their families). Some participants anticipated that MND risk would become salient when their own children came to make reproductive decisions. The relevance of genetic risk in reproductive decisions was contested by others, who did not see it as a factor in their future plans or past decisions.

The salience of risk evolved over time and was mediated temporally, in relation to age, family history, and stage in the life course. As noted earlier, people often imagined they would be affected around the age relatives developed symptoms and approaching this point was accompanied by a sense that ‘this clock is counting down’ (quotation 10, Table 7). While reaching the age at which her mother and grandmother died was ‘a huge shock’, Elaine's worries did not dissipate (‘you can never let that go’). By contrast, Marion (60–69; tested negative), whose mother and brother were both diagnosed at the same age, recalled feeling less worried after reaching this age herself (quotation 11, Table 7). Risk was still a source of worry and preoccupation for younger people, but age could act as a buffer. Thomas (40–49; not tested) said, ‘My mind's clung onto whether I've got it, it'll happen in my 60s. So, I've got 20 years to play with if I lose the coin toss’.

Risk could be more or less salient when family members were living with MND. While some participants described trying to ‘park’ worries to focus on their parent's rapidly changing needs, for Claire, risk remained prominent through her mum's progressing illness (quotation 12, Table 7). At the time of Beverley's (50–59; tested positive) interview, no family members were affected by MND and ‘It's happily lying dormant’. Jasmine (20–29; not tested) felt that if her mum, Jen, was to develop symptoms, ‘that would make the risk level more real’.

### 3.4 ‘Having my time now’: Living well while preparing for the future

Participants employed diverse practices to cope with risk and live well in the face of uncertainty. ‘Getting on with it’ was expressed across interviews. People emphasised staying positive, worrying about it if it happened, focusing on the fact that they were healthy now, and being grateful for what they had (quotation 1, Table 8). Some participants invoked other illnesses and accidents to highlight the unpredictability and uncontrollability of life. In multiple interviews people expressed ‘everyone has to die of something’. Some were fatalistic about the future and took the approach of ‘what will be will be’.

**Table 8:** Illustrative quotations for theme “‘Having my time now’: living well while preparing for the future’.

Quote no.	Illustrative quotation
1	I'd much rather just get on and enjoy the wonderful life I have than dwell too much on it … But I'm afforded that peace because I know what I know about *C9orf72*, because I know that they are trying to stop it. If that wasn't happening, I'm sure I would feel differently … it's a comfort and it's a hope. (Anna, 30–39; tested positive)
2	I'm really keen to invest in experience and to live my life now … enjoy life with our children, wider family and friends, and make sure we maximise those opportunities before any possibility of onset. (Greg, 40–49; not tested)
3	It does govern how I live my life a little bit … just try and do as much as we can … Sometimes it's like, ‘Well, should we really have gone on that holiday? Did we really have the money?’ Hey ho, there's bigger fish to fry … when that point comes, if it comes … I won't have too many regrets. (Thomas, 40–49; not tested)
4	Everybody was very shrugged‐shoulders about it. My brother and me, we were both very worried … My mother‐in‐law was like, ‘Oh, that's ridiculous, you're not going to have it,’ which bothered me a little bit at the time because I needed her support and for her to take it seriously. (Steph, 30–39; tested positive)
5	I'll be much more worried about developing it myself on their [children's] behalf than on mine, because having looked after my mother and having looked after my brother, I don't want them to have to look after me. (Marion, 60–49; tested negative)

A thread running through interviews was a changed outlook orientated towards living life to the full and prioritising the important things (quotations 2 and 3, Table 8). For some, this ‘ethos’ was also shaped by wider experiences of illness and loss. Steph described treating every day as if it was her last—which she saw as a ‘gift’.

It sometimes took ongoing work to keep worries in a ‘manageable place’ (quotation 2, Table 7). Jasmine (20–29; not tested) was happy to support her mum when she wanted to talk about it, but after that ‘put the lid on the box’. For her, ‘I would say that it doesn't affect my life, but that's because I've taken a really hard line in not letting it’. Having family support was important, and it could be difficult when people felt relatives could not understand or did not want to engage with their concerns (quotation 4, Table 8).

Living well also involved undertaking practical strategies to cope with knowledge of risk. Several participants had sought research opportunities, framing this as way to find a positive in their situation and proactively contribute to a treatment for themselves and future generations. For Beverley (50–59; tested positive), ‘I felt like I was taking back some control of my life then, I was doing something about it rather than letting it happen to me’.

While some people did not feel their experiences had impacted their attitudes to work or career, others gave less priority to work and changed jobs to prioritise their quality of life and free time. Susan (50–59; not tested) moved to part‐time work as ‘I just believe in having my time now, just in case’. The risk of iMND also impacted approaches to career progression. Gordon found the possibility of having a long career in front of him hard to imagine and was reluctant to compromise his quality of life to progress professionally. For Maria (50–59; tested positive), avoiding stress was a priority when considering opportunities for promotion. While she initially considered retiring after her positive predictive test, she has since become ‘more measured and calm’, deciding to build up her pension first. Jen retired soon after finding out about her increased MND risk, to enjoy free time and avoid stress. Others valued financial stability, particularly if caring for a family member. Some individuals had been inspired to work in MND or health‐related areas.

Knowing they had an increased chance of developing MND motivated participants to try and maintain health and keep symptoms at bay. People described changing their diet, taking vitamins, avoiding stress, and prioritising health and fitness. Oscar (20–29; not tested) wanted to optimise his health since finding out about iMND in his family: ‘You feel like you need to get that edge’. People sometimes expressed uncertainty around the effectiveness of such changes, but pointed out that ‘it can't hurt’. However, there were those who felt that until there was ‘high quality evidence’, making extreme changes to their diet or exercise routine was not justified.

Although some people took the approach that they would worry about MND if it happened, others found it reassuring to prepare for the possibility of developing symptoms. People described taking out insurance, putting power of attorney in place, and writing a will. Financial planning could be challenging, both emotionally and practically, especially for younger participants who had not anticipated needing to do this so early. Jackie (40–49; not tested) was not sure how to go about taking out insurance or writing a will. Although she sometimes found herself ‘panicking’ about not being prepared, it felt less immediate as her children got older and more independent.

Several participants had thought about the kind of life and death they wanted if they developed MND. Considerations around future housing needs and home adaptations were mentioned by some. The possibility of needing care could be difficult, especially for those who had memories of caring for relatives. People worried about their own children having to witness their health decline and provide care (quotation 5, Table 8). Elaine (50–59; not tested) had thought about her wishes for end of life and completed an Advance Decision. Her approach was ‘plan for the worst, hope for the best’. Jen had discussed assisted dying with her daughter so she knew her wishes.

## 4 DISCUSSION

Our paper highlights the complex ways people made sense of being at risk of MND through integrating various forms of information. It evidences the psychological and emotional impact of living with risk across people's lives, but at the same time suggests that this is not a static experience; rather, its salience fluctuates over time and with changing circumstances. It explores how people sought to cope with this knowledge and live well in the face of uncertainty, and the complex ways they engaged with the possibility of developing symptoms in the future.

Participants made sense of being at risk in multiple ways. Some upheld the chance of inheritance as a random event, privileging a biomedical model, yet in other accounts scientific information interacted with other forms of knowledge. This included family history, which was mapped for patterns and prevalence of the disease, and was often distinctly relational, with perceived risk based in part on identification with affected family members. Our findings thus mirror research on HD, which highlights gut feelings, likeness and proximity to affected relatives as feeding into risk perceptions.^25^, ^26^ Additionally, we found that risk was sometimes perceived as relative, calculated in the context of sibling relationships, as opposed to the logic of independent probability, which is what Wexler^27^ ^,p. Considerations for genetic counseling, para. 8^ evoked when she said, ‘It's difficult to teach someone that “chance has no memory”’. Indeed, Cox and McKellin^25^ argue that risk is rarely understood in terms of Mendelian genetics. Rather than a dichotomy between the biological and social (or scientific vs. unscientific), perceptions can be understood as a ‘complex social calculus of risk’,^25^ ^,p.624^ shaped by abstract knowledge of inheritance patterns, statistics on the average age of onset, alongside how risk *feels*. The translation work involved in making sense of genetic information is articulated by Atkinson et al.^28^ ^,p.1234^:

Genes are not distributed in terms of simple packets of information that are transmitted in clinics and counselling sessions, and which individuals unproblematically receive to become ‘informed’ of their risk … there are processes of translation and interpretation that are brought to bear on genetic information. Background cultural assumptions about inheritance and local assumptions about one's family and kin all interact with professional advice and information.

Uncertainty is a defining feature of risk in MND. While uncertainty may feature in other conditions, in our study it was grounded in the distinct characteristics of MND, and underpinned the narratives of hope woven throughout interviews. One key characteristic that sets MND apart from HD is the incomplete penetrance of MND‐linked genes, as well as the variable age of onset, which left space for hope that it may never happen, and if it did it would be later in life or when treatments had been developed. However, for participants at risk of MND/FTD in Dratch et al.'s^14^ study, uncertainty was related more to *when* they would become sick than *if*, with a sense of ‘dread’ and feeling ‘doomed’ described in interviews— including fear of developing symptoms before treatments were developed. In our study, the potential for future treatments or a cure underlay people's attitudes and decisions, and, as in HD, such hopes were actively cultivated as a way of coping.^29^, ^30^

While a minority of participants did not feel their everyday lives were shaped by genetic risk, a key message of this research is that this knowledge generally had an emotional and psychological impact. In parallel to research by Dratch et al.,^14^ some described knowledge of genetic risk as affecting their views on the self and ability to continue with their lives; uncertainty, while at times seen as a positive, also fed into such challenges. Indeed, mental health issues, trouble sleeping, reckless behaviour and hypervigilance have been evidenced as potential impacts of living with genetic risk across other conditions.^26^, ^31^ Also seen in wider research^14^, ^32^ is how individuals experienced risk as a threat to desired and anticipated future experiences and the ability to fulfil responsibilities to others, which came with a resultant sense of loss. Fear and anxiety over the process of living with and dying from MND were mentioned, grounded in the severity of symptoms people had witnessed in family members. Underscoring meanings of risk in this context was a sense of MND as a uniquely devastating disease.^14^ However, despite the challenges they faced, participants in our research and other studies with a similar population fostered positive aspects from their experiences, describing changed priorities, perspectives, and attitudes to life.^11^, ^12^

While MND risk was described as ‘always in the back of your mind’, a central contribution of this study is in highlighting how its prominence fluctuated. Points where worries came to the forefront included when noticing possible symptoms; around predictive testing; in the context of reproduction; and in relation to various temporal factors including disease‐related events in the family. Thus, we found that fluctuations occur in everyday life (e.g., when tripping over or dropping something), when there are triggers, and change with age/across the life course. An ebb and flow of risk salience has been described in research on other genetic conditions,^25^, ^33^, ^34^ which outlines interwoven social, biographical and temporal factors which bring risk into the ‘here and now’,^25^ where it is perceived as problematic and proximate. Kenen et al.'s^34^ concept of ‘chronic risk’ encapsulates this important aspect of the risk experience—that knowledge of MND risk is biographically disruptive,^35^ in that it may entail changes in behaviours, social relationships and self‐identity, unsettling taken‐for‐granted views on self, biography and the future. At the same time, worries of the disease came to prominence at critical junctures and at other times remained in the background at a low zone of relevance.^36^, ^37^

People described diverse and evolving approaches and strategies to cope with risk, often putting in considerable work to maintain it in the back of their minds and live well in the present. Indeed, our research reflects wider risk literature in highlighting examples of avoidance, compartmentalisation, fatalism or resignation, or normalising risk against other uncontrollable diseases or events.^30^, ^34^, ^38^, ^39^ Our paper also explores the complex ways people engaged with the possibility of developing symptoms. This, we found, involved working to maintain health, yet also preparing for the possibility of developing symptoms. Aspects such as adapting diet and trying to avoid ‘triggering’ the gene are similarly paralleled in wider literature on variably penetrant conditions^34^, ^40^ ‐ yet our research found varied attitudes towards how far people were willing to modify their lifestyle based on mixed or limited evidence.

Considering these findings, there is an urgent need for dedicated and tailored support for those living with genetic risk of MND. This is needed regardless of whether a genetic cause has been identified in the family or confirmed through predictive testing, as significant emotional and psychological impacts were reported across interviews irrespective of these factors. Another important finding is that the salience of risk fluctuates over time and with changing circumstances, pointing to the need for support to be open‐ended to meet people's changing needs. We have also touched upon the diverse approaches and strategies people employ to cope and live well with knowledge of MND risk. The above findings suggest participants valued having people to talk to about their worries (in professional and informal contexts); receiving empathetic and timely investigations when they had concerns over possible symptoms; and in some cases, having ongoing monitoring from healthcare professionals. Future research should explore the forms support could take and focus on developing research‐informed resources and interventions.

While approaches to seeking information varied, reliable and accessible information should be available for those who want it, including on genetics in MND, its clinical features and management, and research developments. However, helping people understand their risk (where this knowledge is wanted) is not just about tackling misunderstandings and knowledge gaps, but rather being mindful of the complex logic involved in making sense of genetic information. People may know they objectively have a 50% risk, but that does not mean risk is made sense of through purely scientific and rational discourse. This is an important message for healthcare professionals working with families affected by MND, including genetic counsellors. This study has suggested that research participation can be valuable to people in that it allows them to act against the disease and enact hope for the future, thereby supporting coping mechanisms.

It should be noted that since this study was conducted, developments in research and trials have evolved. Introducing additional complexities, uncertainties and hopes, this has the potential to shape meanings around genetic risk. Interviewing offers an in‐depth exploration of a person's perspectives and experiences^21^ at a particular point in time, underscoring the need to understand how people make sense of and incorporate risk into their lives and decisions as trials and treatments evolve. While a range of perspectives were explored, certain demographic groups are underrepresented and warrant further research.

## 5 CONCLUSIONS

This study explored experiences of living with genetic risk of MND, from the multiple vantage points of those with a variety of family experiences and genetic testing decisions and outcomes. While living with genetic risk impacted people in different ways, the salience of this knowledge fluctuated. Experiences were characterised by multiple uncertainties that shifted over time. Understanding the issues and decisions faced by families affected by iMND is pertinent given the unmet information and support needs that have been identified in previous studies^11^, ^12^, ^41^ and reinforced here. Findings from this study have been used to develop a resource on iMND on the healthtalk.org website to help address these needs.

## AUTHOR CONTRIBUTIONS

**Jade Howard**: Funding acquisition; writing—original draft; methodology; writing—review and editing; formal analysis; project administration; investigation; conceptualisation. **Fadhila Mazanderani**: Conceptualisation; funding acquisition; writing—review and editing; methodology; formal analysis; supervision; project administration. **Karen Forrest Keenan**: Conceptualisation; methodology; writing—review and editing; formal analysis; supervision; Project administration. **Martin R. Turner**: Conceptualisation; funding acquisition; writing— review and editing; methodology; Formal analysis. **Louise Locock**: Conceptualisation; funding acquisition; writing—review and editing; methodology; formal analysis; supervision; project administration.

## CONFLICT OF INTEREST STATEMENT

Louise Locock declares that she became a member of the MND Association's Health Research Advisory Panel in 2021. The remaining authors declare no conflict of interest.

## ETHICS STATEMENT

Ethics approval for this study was granted by the Berkshire Ethics Committee (REC Ref 12/SC/0495). Participants consented to take part in the interview and gave a copyright for their material to be used in publications.

## Data Availability

Deidentified data are available under licence from the University of Oxford. All data requests should be submitted to hergadmin@phc.ox.ac.uk for consideration.
